# Hypofractionated carbon‐ion radiotherapy for stage I peripheral nonsmall cell lung cancer (GUNMA0701): Prospective phase II study

**DOI:** 10.1002/cam4.2561

**Published:** 2019-09-18

**Authors:** Jun‐ichi Saitoh, Katsuyuki Shirai, Tatsuji Mizukami, Takanori Abe, Takeshi Ebara, Tatsuya Ohno, Koichi Minato, Ryusei Saito, Masanobu Yamada, Takashi Nakano

**Affiliations:** ^1^ Gunma University Heavy Ion Medical Center Maebashi Gunma Japan; ^2^ Department of Radiation Oncology Faculty of Medicine University of Toyama Toyama Toyama Japan; ^3^ Department of Radiology Saitama Medical Center Jichi Medical University Saitama Japan; ^4^ Department of Radiation Oncology Gunma Prefectural Cancer Center Ota Gunma Japan; ^5^ Department of Radiation Oncology School of Medicine Kyorin University Mitaka Tokyo Japan; ^6^ Department of Respiratory Medicine Gunma Prefectural Cancer Center Ota Gunma Japan; ^7^ Department of Respiratory Medicine National Hospital Organization Shibukawa Medical Center Shibukawa Gunma Japan; ^8^ Department of Medicine and Molecular Science Gunma University Graduate School of Medicine Maebashi Gunma Japan

**Keywords:** carbon‐ion radiotherapy, heavy ion radiotherapy, nonsmall cell lung cancer, phase II clinical trial, prospective study

## Abstract

This phase II study's aim was to confirm the efficacy and safety of hypofractionated carbon‐ion radiotherapy in patients with stage I peripheral nonsmall cell lung cancer (NSCLC). The study encompassed 37 patients with histologically proven peripheral stage I NSCLC in the period June 2010‐March 2015. All underwent the planned full dose of carbon‐ion radiotherapy, administered with relative biological effectiveness of 52.8 Gy and 60 Gy (divided into four fractions over 1 week) for T1 and T2a tumors, respectively. The 2‐year local control rate was set as the primary endpoint, while overall survival, progression‐free survival, and the incidence rates of acute and late adverse events were secondary endpoints. The patients were followed up for 56.3 months overall and 62.2 months in the surviving patients, respectively. The actuarial local control rates were 91.2% after 2 years, and 88.1% after 5 years. No differences were found between the T1 and T2a tumors in the 5‐year local control rate (90.9% vs 86.7%, *P* = .75). The actuarial overall survival rates achieved 91.9% for 2‐year and 74.9% for 5‐year period. T1 tumors showed actuarial 5‐year overall survival rates of 80%, compared to 66.7% in T2a tumors. Two patients with T2a tumors and either severe emphysema or bronchiectasis experienced lung toxicity ≥ grade 2, in contrast to T1 patients who only experienced mild toxicities (lower than grade 2). The findings suggest that carbon‐ion radiotherapy is effective and safe for peripheral stage I NSCLC; however, further clinical evaluations are needed to confirm its therapeutic efficacy.

Trial registration: UMIN000003797. Registered 21 June 2010, prospectively registered.

## Graphical abstract

The aim of this phase II study was to confirm the feasibility and safety of hypofractionated carbon‐ion radiotherapy for patients with stage I peripheral nonsmall cell lung cancer. The actuarial 2‐year and 5‐year local control rates were 91.2% and 88.1%, respectively. The actuarial 2‐year and 5‐year overall survival rates were 91.9% and 74.9%, respectively.

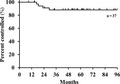

## 1 INTRODUCTION

Lobectomy and mediastinal lymph node dissection represent the current standard of treatment for stage I NSCLC.^1^ Nevertheless, radical surgery is contraindicated for patients with severe comorbidity or poor pulmonary function. In those patients, stereotactic body radiation therapy with photons (SBRT) may provide an alternative choice of treatment. A multi‐centric prospective study employing SBRT for treating NSCLC (T1N0M0) reported comparable 3‐year overall survival rates for the operable and inoperable populations, of 76.5% and 59.9%, respectively; however, there was a relatively high incidence of severe lung toxicity, especially among the inoperable patients.^2^

Compared with photons, carbon ions can provide an increased probability of tumor control because of their higher linear energy transfer as well as greater relative biological effectiveness (RBE).^3^, ^4^ In addition, the Bragg peak and small lateral scattering of carbon ions result in a theoretically superior dose distribution to that of photons. A prospective phase I/II study at the National Institute of Radiological Sciences Hospital (Chiba, Japan) dealing with application of carbon‐ion radiotherapy (C‐ion RT) for treating stage I NSCLC used fixed doses of 52.8 Gy and 60 Gy (RBE) for T1 tumors and T2 tumors, respectively, delivered in four fractions over 1 week.^5^ It was shown that the overall 5‐year local control rate was 90%, while 97% and 80% were achieved for T1 and T2 tumors, respectively.^5^ A dosimetric analysis comparing C‐ion RT and SBRT for stage I peripheral NSCLC reported that the dose distribution of C‐ion RT exhibited better target conformity and spared normal lung tissue as well as tissues of trachea, esophagus, heart, and spinal cord.^6^ However, there have been few prospective assessments of C‐ion RT efficacy for the stage I NSCLC at other C‐ion RT facilities. We therefore undertook a prospective phase II study to confirm the therapeutic efficacy and safety of C‐ion RT in patients with the stage I NSCLC.

## 2 MATERIALS AND METHODS

### 2.1 Patients

Here, we considered the following eligibility criteria for patients: histologically proven peripheral stage I NSCLC, diagnosed in line with TNM Classification of the Union for International Cancer Control's (7th Edition); inoperable, or refusal of surgery; a measurable tumor; and an Eastern Cooperative Oncology Group's scale performance status between 0 and 2. The following were set as exclusion criteria: a previous history of radiotherapy anywhere near the target volume; chemotherapy within the month prior to C‐ion RT; a life expectancy estimated to be ≤6 months; a second active cancer; interstitial pneumonitis; or an intractable infectious disease in the region of the target volume. Each patient's eligibility was confirmed at a joint conference involving medical oncologists, thoracic surgeons, and radiation oncologists.

The study was conducted in line with the Declaration of Helsinki. The ethics committee approved the trial, and all patients gave written informed consent. The trial was registered under the number UMIN000003797 at the University Hospital Medical Information Network Center (http://www.umin.ac.jp).

### 2.2 Primary and secondary endpoints

Two‐year local control rate was selected as the primary endpoint, whereas the rates of overall survival (OS), progression‐free survival (PFS), and incidence rate of acute and late adverse events were chosen as secondary endpoints.

### 2.3 Planning and treatment

Depending on the tumor location, the patient was immobilized in the supine or prone position using a thermoplastic shell (Shellfitter; Sanyo Polymer Industrial) with a pillow made of water‐sclerogenic polymers (Moldcare; ALCARE). To achieve a suitable posture for oblique beam irradiation, the patient was rotated ± 15° to the superior‐inferior axis. Subsequently, computed tomography (CT) was run in 2‐mm slices with two different body positions. A respiratory‐gated CT image was obtained after exhaling. This was followed by a four‐dimensional CT scan to account for respiratory motion, reconstructing four‐dimensional images for each phase of respiration.

For the treatment, for respiratory‐gated irradiation the gating level was set to within 30% of the wave height around peak exhalation. The following volumes were defined in the lung window: the gross tumor volume (GTV), the clinical target volume (CTV), and the planning target volume (PTV). CTV was defined as the GTV dilated 5 mm into the pulmonary parenchyma. The internal margin was set considering respiratory movement in each direction, as determined from the four‐dimensional CT, with a 3‐mm setup margin. Finally, to create PTV, a planning margin calculated as the square root of the sum of squares of the internal and setup margins was added to the CTV. The clinical dose distribution was calculated based on physical dose and RBE, in line with the previous results.^3^ XiO‐N treatment planning software (Elekta/ Mitsubishi Electric) was used to calculate the passive scattering carbon‐ion dose distribution. The prescribed dose (RBE) of C‐ion RT for T1a and T1b tumors was 52.8 Gy, whereas 60.0 Gy dose was chosen for T2a tumors, in both cases divided to four fractions over 1 week. The dose was administered to the PTV's isocenter.

The percentage of the normal lung volume receiving more than 20 Gy (lung's V20) was set not to exceed 20%. Although the dose constraints for esophagus, trachea and main bronchus, spinal cord, and heart were not clearly established, the volumes of 20Gy (RBE) were suppressed as low as possible based on our institutional policy.

### 2.4 Patient and tumor response evaluations

Prior to treatment, the patients underwent the following evaluations: blood cell and biochemical analyses (blood cell counts, differential counts, serum biochemistry), electrocardiography, pulmonary function tests, imaging (chest radiography, CT of the thorax and abdomen, whole‐brain CT or magnetic resonance imaging, and ^18^F‐fluorodeoxyglucose positron emission tomography. During 6 months following the C‐ion RT, the patients underwent a physical examination, toxicity assessments, and X‐ray of the chest every month, while thoracic CT scan with blood analyses were obtained every 3 months. The tumor response to the applied therapy was evaluated in line with the version 1.1 of the Response Evaluation Criteria in Solid Tumors guidelines.^7^ Toxicity was assessed in line with the version 4.0 of the Common Terminology Criteria for Adverse Events.

### 2.5 Statistical analysis

We calculated local control rates or survival times starting with the first day of C‐ion RT administration. The Kaplan‐Meier method was applied to determine the defined endpoints (local control rate, OS rate, and PFS rate). For purposes of sample size calculation, we hypothesized that the 2‐year local control rate of stage I NSCLC would be 60% by conventionally fractionated radiation therapy with photons and 90% by C‐ion RT. Using the normal approximation, we calculated that 35 patients would be needed for 80% power and 95% confidence. In the univariate analyses, we applied log‐rank tests to evaluate the effects of age group, sex, histological type, performance status, T stage of TNM classification, operability, and smoking history on the study's endpoints. *P*‐values of lower than 0.05 were considered as statistically significant. All tests were performed two‐tailed with JMP version 12.2.0 (SAS Institute Inc).

## 3 RESULTS

### 3.1 Patients

Between June 2010 and March 2015, 37 patients satisfying all inclusion and exclusion criteria (25 men and 12 women; median age of 73 years with age range of 47‐85 years) were recruited. All patients were followed up at least 2 years or until death, with the median follow‐up duration of 56.3 months for all patients and 62.2 months for the surviving patients. The Table 1 shows a summary of characteristics of the patients and tumors. In 24 patients, the tumor was in the T1 stage while T2a was recorded in 13 individuals. In terms of histological types there were 24 patients with adenocarcinoma and 13 with squamous cell carcinoma. Nine of 37 patients were judged to be inoperable.

**Table 1:** Patient and tumor characteristics

Number of patients		37
Sex	Male/Female	25/12
Age (years)	Median	73
	Range	47‐85
Performance status	0/1/2	17/18/2
Tumor site	Upper lobe	23
	Middle lobe	2
	Lower lobe	12
Tumor classification	T1a/T1b/T2	12/12/13
Histology	Adenocarcinoma	24
	Squamous cell carcinoma	13
Operability	Yes/No	28/9
Smoking	Yes/No	20/17
Planning target volume (mL)	Median	56.9
	Range	18.1‐119.2

### 3.2 Dosimetric analysis

**Figure 1:**
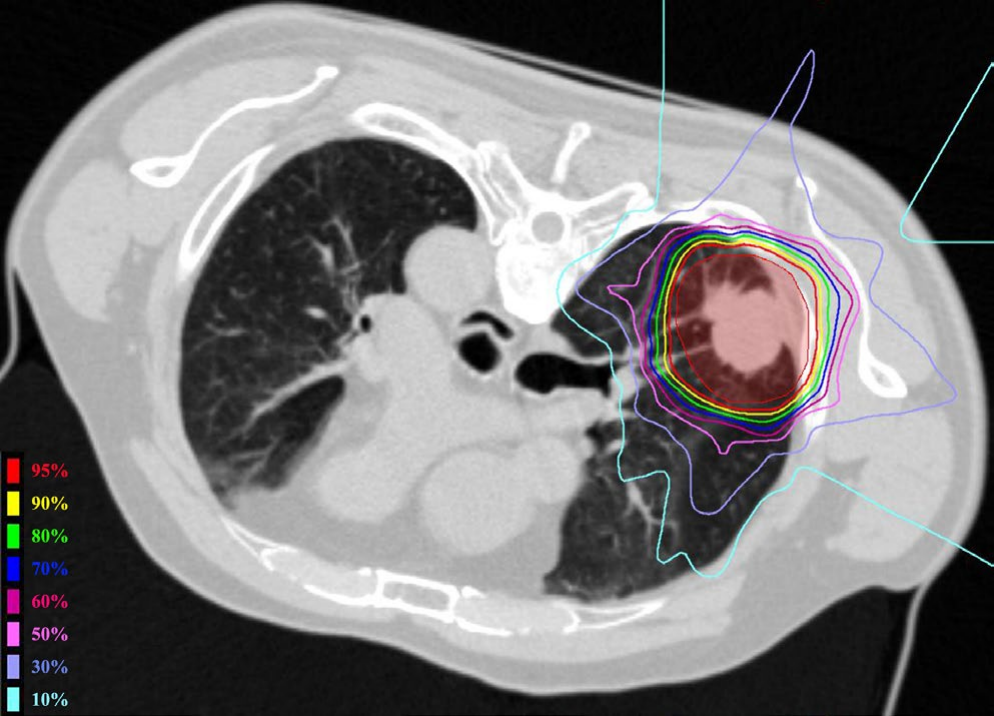
Dose distribution of carbon‐ion radiotherapy in a patient with T2 tumor. PTV was painted red. The percentage of the normal lung volume receiving more than 20 Gy was 8% in this patient

All patients received the planned full dose of C‐ion RT. The median percentage of dose received by more than 95% of the PTV relative to the isocentric dose was 96.0% (range, 80.1%‐99.8%). The median of lung's V20 was 5.7% (range, 1.4%‐10.6%). The delineation of the PTV and dose distribution of a representative case with axial image is shown in Figure 1.

### 3.3 Local control and survival

**Figure 2:**
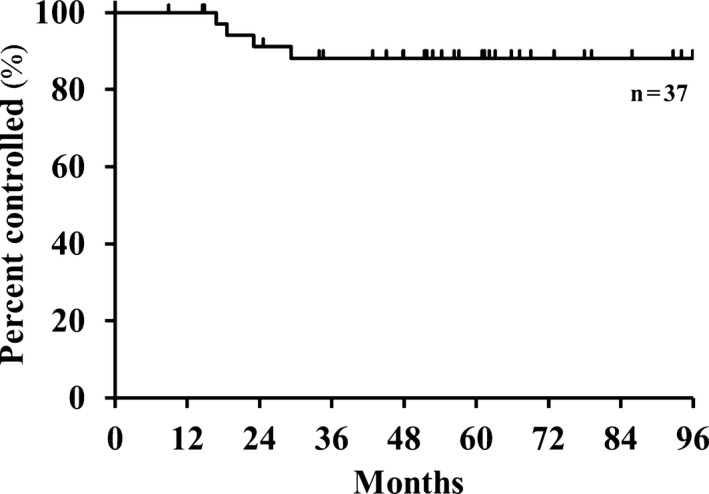
Local control rate during the follow‐up period (N = 37)

Table 2 summarizes the failure patterns. Four patients experienced local recurrence. The local control rate throughout the duration of the follow‐up is plotted in Figure 2. The values of actuarial 2‐year, 3‐year, and 5‐year local control rates were 91.2% (95% confidence interval [CI], 76.0‐97.1), 88.1% (95% CI, 72.3‐95.5), and 88.1% (95% CI, 72.3‐95.5), respectively. For the patients with T1 tumors, the 2‐year and 5‐year local control rates were 91.3% and 86.7%, similar to the patients with T2a tumors where both local control rates achieved 90.9% (*P* = .75). Smokers exhibited lower 2‐year local control rates than nonsmokers (82.4% vs 100.0%, *P* = .03). The 2‐year local control rates were 90.0% and 91.2% for patients with squamous cell carcinoma and those with adenocarcinoma, respectively (*P* = .87). There were no significant associations between local control rates and any of the other clinical factors. Two patients experienced local‐only progression; they underwent salvage treatment by surgery or reirradiation with C‐ion RT, and both survived without disease reprogression more than 3 years after this treatment.

**Table 2:** Analysis of the failure patterns for the T1 and T2 tumors

Failure pattern	T1	T2
Local only	2	0
Local and regional	1	0
Local and distant	0	1
Local, regional, and distant	0	0
Regional only	0	1
Regional and distant	4	1
Distant only	2	1

**Figure 3:**
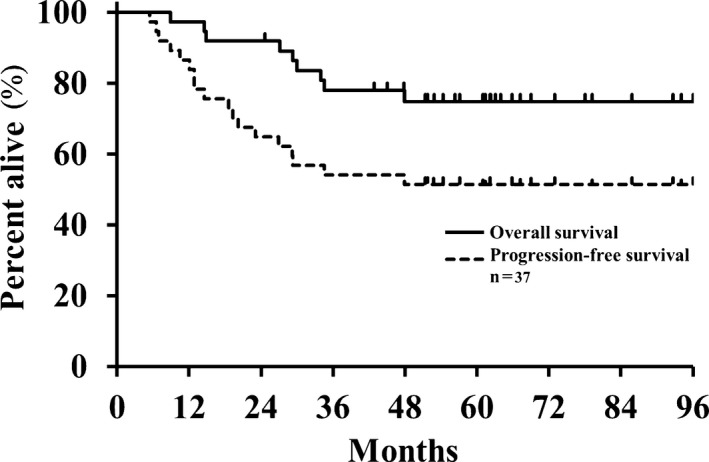
Overall (solid line) and progression‐free (dashed line) survival from the start of treatment (N = 37)

The OS and PFS rates throughout the follow‐up period are plotted in Figure 3. During follow‐up, four patients died of disease progression and five of intercurrent diseases. The actuarial 2‐, 3‐, and 5‐year OS rates achieved 91.9% (95% CI, 77.7‐97.4), 80.0% (95% CI, 61.7‐88.6), and 74.9% (95% CI, 58.2‐86.4), respectively. The 5‐year OS rates for the various subgroups were as follows: 80.0% for individuals with T1 tumors and 66.7% for those with T2a tumors (*P* = .39); 84.7% for the operable vs 44.4% for inoperable patients (*P* = .005); 53.9% for patients with squamous cell carcinoma compared with 86.7% for those with adenocarcinoma (*P* = .01); and 58.4% for smokers; and 93.8% for nonsmokers (*P* = .01). The 5‐year OS rates showed no significant associations with the other clinical factors.

Thirteen patients experienced disease progression. The actuarial 2‐year, 3‐year, and 5‐year PFS rates were 64.9% (95% CI, 48.5‐78.4), 54.1% (95% CI, 38.1‐69.2), and 51.4% (95% CI, 35.6‐66.8), respectively. In addition to the two patients described earlier who received salvage therapy for local progression, three patients underwent salvage treatment by C‐ion RT or stereotactic radiotherapy for regional lymph node metastases or solitary brain metastasis. The 5‐year PFS rates were 52.0% and 50.0% for the T1 tumors and T2a tumors, respectively (*P* = .74), and 25.0% for smokers and 82.4% for nonsmokers, respectively (*P* = .0003). The 5‐year PFS rates showed no significant associations with the other clinical factors.

### 3.4 Assessment of toxicity

Table 3 summarizes the incidence of acute and late adverse events. No patients experienced toxicity with severity classified as grade 4 or higher. One patient with severe emphysema developed grade 3 pneumonitis, and another with bronchiectasis and atypical mycobacteriosis developed grade 2 pneumonitis. Pneumonitis of grade 2 severity or higher showed a cumulative incidence of 5%. Both these patients had T2a tumors; no patient with a T1 tumor experienced toxicity of severity greater than grade 1.

**Table 3:** Incidence of acute and late toxicity

Toxicity grade	Number of patients (N = 37)					
	0	1	2	3	4	5
Blood	21	16	0	0	0	0
Lung	1	34	1	1	0	0
Skin	12	23	2	0	0	0
Rib	31	6	0	0	0	0
Esophagus	37	0	0	0	0	0
Heart	37	0	0	0	0	0

## 4 DISCUSSION

In this trial, the local tumor control rate was selected as the primary endpoint. The 2‐year and 5‐year local control rates measured 91% and 88%, respectively, very similar to those reported of a previous phase II study of C‐ion RT (90% and 90%, respectively).^5^ In our study, local control rate did not differ significantly between the tumors in stage T1 and T2a. Compared to the previous study,^5^ there was greater local recurrence of T1 tumors in the present study but less recurrence of T2 tumors.

The local recurrences may have been the result of a problem with the robustness of the treatment planning. We previously analyzed and reported some factors related to the possibility of dose degradation,^8^, ^9^, ^10^ as well as proposing some technical measures to improve the robustness of the planning.^11^ Following this analysis, we installed CT in the C‐ion RT treatment room at our facility to allow accurate positioning and dose verification. Another explanation for the recurrence may be the smaller dose administered for T1 tumors, which was less than that for T2a tumors. Following the present study, for T1 tumors treated at our facility we have increased the dose to 60.0 Gy (RBE) in four fractions, since this dose has been shown here to be safe for patients with tumors >3 cm. Furthermore, a dose escalation study of single‐fraction C‐ion RT for stage I NSCLC conducted in Japan demonstrated the efficacy and safety of a single fraction of 48‐50 Gy (RBE).^12^

SBRT provides superior tumor control than conventional radiotherapy in treating stage I lung cancer, but few studies of SBRT reported the local tumor control over long‐term follow‐up. In three studies, the 5‐year local control rates with SBRT were 78%‐92% for T1 tumors and 69%‐73% for T2 tumors.^13^, ^14^, ^15^ A large cohort study reported 84% 3‐year local control rate in T1 tumors, compared to 74% for T2.^16^ Typically, 10%‐20% lower local control rate with SBRT was reported for T2 tumors than for T1. A Japanese dose escalation phase I study of SBRT with 40 Gy starting dose and 5 Gy increments was undertaken to improve local control of T2N0M0 NSCLC (>3 cm) tumors.^17^, ^18^ The report recommended a dose of 55 Gy in four fractions for peripheral PTVs < 100 cm^3^, and 50 Gy in four fractions for larger tumors with PTVs > 100 cm^3^.^17^, ^18^ Shibamoto et al published the long‐term results of SBRT treatment using a radiobiology‐based regimen.^14^ The prescribed doses of SBRT were 44‐48 Gy and 52 Gy for T1 and T2 tumors, respectively, administered in four fractions with interfraction intervals of at least 3 days. The rate of local control for the T2 tumors was 73%, similar to that in previous reports of SBRT, although it should be noted that there was a relatively higher incidence of higher grade pneumonitis (21%).^14^ In the present C‐ion radiotherapy trial, the 5‐year local control rate reached 90.9% for T2a tumors, suggesting that the regimen of 60 Gy in four fractions was effective and possibly superior to that of SBRT. However, the number of treated patients was relatively small, so further evaluation is needed to confirm the efficacy of this treatment for T2 tumors.

Several previous studies have reported 2‐year or 3‐year OS rates following SBRT, with 3‐year OS rates reported as 40%‐60%.^16^, ^19^, ^20^, ^21^ A meta‐analysis of SBRT studies published in 2011 reported pooled estimates of 3‐year OS of 56% and 5‐year OS of 36%, respectively.^22^ OS is typically affected by late recurrence or intercurrent diseases, so it should be evaluated over a long follow‐up period. Here, we found 80.0% 3‐year OS and 74.9% 5‐year OS rates, with 62.2 months duration of follow‐up for the surviving patients.

There are various possible explanations for the favorable OS in our study. One relates to the patients' condition and the evolution of salvage treatment. In our study, 76% (28/37) of the patients were considered to be operable, and two patients with local recurrence and three patients with regional lymph node recurrence or brain oligometastasis underwent salvage treatment by surgery, C‐ion RT or stereotactic radiotherapy. Otaki et al reported no severe complications, perioperative death, or local recurrence after salvage surgery following C‐ion RT, although combined resection was sometimes needed because of severe adhesion.^23^ A second reason for the favorable OS in our study may be related to histological type of NSCLC, considering that in this study OS was better in the patients with adenocarcinoma histological type. Indeed, three patients with adenocarcinoma experienced slow‐growing lung metastases and survived the follow‐up period without any treatment.

SBRT of lung tumors may lead to lung toxicity, which is dose‐limiting effect. In a meta‐analysis of SBRT studies it was reported that the incidence of grade 3 to 5 adverse events was 8%, with the incidence increasing among patients treated with a higher biologically effective dose.^22^ The RTOG 0236 trial in the USA tested 60 Gy in three fractions and showed a 16% rate of grade 3 and grade 4 lung toxicity.^24^ Recent reports regarding SBRT have reported the incidence of lung toxicity at grade 2 or higher as 1%‐13%.^2^, ^13^, ^14^, ^15^, ^16^, ^19^ In the previous phase II study of C‐ion RT, grade 2 lung toxicity was seen in 1% of the patients, and there were no toxic reactions of grade ≥3.^5^ None of our patients with tumor in T1 stage experienced pneumonitis ≥grade 2. Grade 3 pneumonitis was recorded in only one patient with a T2 tumor; this patient had been considered inoperable because of severe lung emphysema. In the Japanese prospective trial, lung toxicity ≥grade 3 was reported in 6% of operable patients and 13% of inoperable patients.^2^ In the dosimetric analysis that compared C‐ion RT and SBRT, described earlier, the lung's V20 with C‐ion RT was about 50% of that with SBRT treatment, and the normal lung volume receiving ≥5 Gy was one‐third that for SBRT.^6^ C‐ion RT may therefore be considered as an effective and safe option for treating the patients with larger or inoperable tumors. In addition, severe adverse events in organs at risks without lung were not observed at all. Treatment target in this study was peripheral stage I NSCLC, and the mediastinal components were not included in the high‐dose irradiated areas in all patients. More precise evaluation about the dose volume statistics for each organ at risk in hypofractionated C‐ion RT will be confirmed in the future study.

In this study, smoking history significantly affected the local control after C‐ion RT. There were few reports about the difference of local control after radiation therapy between smokers and nonsmokers. In the analysis of postoperative radiation therapy with photons for NSCLC, Nguyen et al reported that smokers had worse 5‐year local control than nonsmokers (70% vs 90%, *P* = .001), and histology was not the significant factor on univariate analysis for local control.^25^ They assumed that the harmful effects of smoking during radiation therapy may be explained by its hypoxic effect. In another report of prognostic factors in patients with glottis cancer treated with photon radiation therapy, local control was negatively affected by tobacco smoking (particularly more than 20 cigarettes per day over a period of time—more than 40 pack‐years).^26^ The mechanisms underlying effects by smoking on C‐ion RT remain uncertain, and the results might be confirmed in further study.

The present study had some limitations. It was a single‐center prospective phase II study at a single center, and the number of participants was limited. Since April 2016, a multicenter prospective registry study of Japanese C‐ion RT facilities has been established; this applies an integrated treatment schedule for treating stage I peripheral NSCLC, administering four fractions over 1 week to provide a 60 Gy dose of (RBE). This prospective registry study is expected to demonstrate further improvements in treatment results for patients with stage I NSCLC.

Our prospective study confirmed that C‐ion RT was effective and safe in the treatment of stage I peripheral NSCLC. We considered that this study could show the validation about RBE assumption in the treatment planning of carbon beam between the different facilities, since the clinical results were almost same as the previous report. Our findings support the need for further clinical evaluations of the therapeutic efficacy of this treatment, which needs to be confirmed by a multicenter prospective registry study of Japanese C‐ion RT facilities.

## CONFLICT OF INTEREST

The authors declare that they have no competing interest.
